# Supplementary material for: MNS induces antiviral protection and suppresses inflammation
Source: Ann Med. 2026 May 14;58(1):2662146. doi: 10.1080/07853890.2026.2662146 (PMC13178039; doi:10.1080/07853890.2026.2662146)
Supplement: Supplemental Material [file IANN_A_2662146_SM6382.docx]

**Supplementary Figure 1.**

(A) Dose-response curves showing the concentration-dependent inhibition of viral replication by MNS. Half-maximal effective concentrations (EC50) were calculated by nonlinear regression analysis. (B) Cell viability of RAW 264.7 cells and HT1080 cells treated with increasing concentrations of MNS in the absence of viral infection, assessed using a luminescence-based viability assay. Half-maximal cytotoxic concentrations (CC50) were determined by nonlinear regression. Data are mean ± SEM; N.S., p > 0.05; *p < 0.05, **p < 0.01, ***p < 0.001.

**Supplementary Table 1. Primer sequences used for RT-qPCR.**

Forward and reverse primer sequences (5'→3') for all genes analyzed in this study.
